# Mapping Fos-immunoreactive neurons activated by intra-oral infusion of quinine, sucrose or water throughout the brain of B6 mice

**DOI:** 10.1007/s00429-026-03095-8

**Published:** 2026-03-21

**Authors:** Michael S. King, Lianyi Lu, Max L. Fletcher, John D. Boughter

**Affiliations:** 1 Department of Biology, Stetson University, DeLand, FL USA; 2 Department of Anatomy and Neurobiology, The University of Tennessee Health Science Center, 331 Wittenborg Bldg., 855 Monroe Ave, Memphis, TN 38163 USA

**Keywords:** Amygdala, Gustatory, Nucleus of the solitary tract, parabrachial nucleus, Reticular formation, Taste

## Abstract

Fos immunohistochemistry was used to identify neurons in taste-related brain areas throughout the B6 mouse brain activated by intra-oral (IO) infusion of 3.0 mM quinine hydrochloride (Q), 1.0 M sucrose (S) or filtered water (W). IO infusion of Q and S elicited more Fos-immunoreactive (Fos-IR) neurons than W in the central medial (CM) and dorsomedial (DM) subareas of the parabrachial nucleus (PBN) and the central medial (CeM) amygdala (ps < 0.05). Infusion of Q led to more Fos expression than W in the central lateral (CL) PBN and the parvocellular reticular formation (PCRT; ps < 0.05). The only area where IO infusion of Q and S elicited a different number of Fos-IR neurons was the PCRT which responded more to Q (*p* < 0.05). Cluster analysis of the number of Fos-IR neurons in all 29 taste-related nuclei and subareas examined revealed that populations of neurons distributed among these brain regions respond best to Q, S or both Q and S. Specifically, the Q-best cluster included more posterior structures like the nucleus of the solitary tract, RT and part of the PBN. The S-best cluster included more anterior structures like the bed nucleus of the stria terminalis, nucleus accumbens and orbitofrontal cortex. And, the cluster of areas that responded better to Q and S than W included the amygdala, gustatory and piriform cortices and a few PBN subareas. Therefore, the data suggest that collections of neurons among taste-responsive brain areas are important for distinguishing Q and S from water as well as identifying the specific tastant.

## Introduction

Taste receptors embedded within the oral epithelium respond to chemicals introduced into the oral cavity and this information is carried to the brain by the facial, glossopharyngeal, and vagus nerves (reviewed in Lundy and Norgren 2015). The first nucleus in the brain to receive taste input, as well as other orosensory input, is the rostral nucleus of the solitary tract (NST) in the medulla. In rodents, the ascending taste pathway then proceeds to the parabrachial nucleus (PBN), gustatory thalamus (GT) and the gustatory cortex (GC). The NST is split into several subareas (reviewed in King 2007) including the central (C), which is the main recipient of afferent taste input and source of the projection to the PBN, the lateral (L), which contains neurons that primarily respond to orotactile input (Travers and Norgren 1995), the medial (M), which contains preganglionic parasympathetic neurons that control salivation (Contreras et al. 1980), and the ventral (V), which contains pre-motor neurons that project to the subjacent reticular formation (RT; Halsell et al. 1996; Ganchrow et al. 2014). The reticular nucleus or formation (RT) is split into several portions including parvocellular (PCRT) and intermediate (IRT) that coordinate oromotor output (Travers et al. 1997). The PBN contains several subareas with distinct cytoarchitecture and connectivity (Fulwiler and Saper 1984). Most of the ascending gustatory pathway originating in the rostral NST terminates in the waist region of the caudal PBN that includes the central medial (CM) and ventral lateral (VL) subareas (Halsell and Frank 1991; Tokita and Boughter 2016). Some taste input also is received by the external medial (EM) and external lateral (EL) subnuclei of the PBN (Yamamoto et al. 1994; Halsell and Travers 1997). Other subareas of the PBN include the central lateral (CL), dorsal medial (DM) and dorsal lateral (DL), the latter of which contains neurons that respond to IO infusion of sugars (Yamamoto et al. 1994; Yamamoto and Sawa 2000; Tokita et al. 2014). The main ascending orosensory pathway proceeds from the waist area and EL of the PBN to the GT, (part of the ventral posteromedial nucleus of the thalamus), and then to the GC in the insula (reviewed in Lundy and Norgren 2015; Staszko and Boughter 2021). Several of the other nuclei targeted in the current study have subareas (Table 1) that are interconnected with these main components of the taste pathway and play a variety of roles in taste-related behaviors and learning (Krukoff et al. 1993; Whitehead et al. 2000; Lundy and Norgren 2004; Smith et al. 2005; Tokita et al. 2009, 2010; Szabó et al. 2018; Samuelsen and Vincis 2021; Idris et al. 2023).

**Table 1:** Number of sections and subnuclei for each brain area examined

Brain area	Number of sections	Subnuclei	References
Rostral NST	4	M, C, L, V	Ganchrow et al. 2014
RT	4 (same as NST)	PCRT, IRT	Travers et al. 1997
PBN	7	CM, VL, DM, CL, DL, EL, EM	Tokita et al. 2009 Tokita et al. 2010
IC	2		
GT	3		
GC	5	GI, DI, AI	Cechetto and Saper 1987
PIR	5 (same as GC)		
AM	4	LA, BL, CeL, CeM	
LH	4 (same as AM)		
OFC	3	LO, VO, MO	
NAc	4	Core, shell	
BNST	4		
PV thalamus	2		

AI, agranular insula; AM, amygdala; BL, basolateral amygdala; BNST, bed nucleus of the stria terminalis; C, central; CeL, central lateral; CM, central medial; CL, central lateral; CM, central medial; DI, dysgranular insula; DL, dorsal lateral; DM, dorsal medial; EL, external lateral; EM, external medial; GI, granular insula; GC, gustatory cortex; GT, gustatory thalamus; IC, inferior colliculus; IRT, intermediate reticular formation; L, lateral; LA, lateral amygdala; LH, lateral hypothalamus; LO, lateral orbitofrontal; M, medial; MO, medial orbital; NAc, nucleus accumbens; NST, nucleus of the solitary tract; OFC, orbitofrontal cortex; PBN, parabrachial nucleus; PCRT, parvocellular reticular formation; PIR, piriform cortex; PV, paraventricular; RT, reticular formation; V, ventral; VO, ventral orbital

*Paxinos and Franklin 2001 was used to delineate all brain areas

There is evidence that intra-oral (IO) infusion of different taste solutions activates different neuron populations in the NST (Harrer and Travers 1996; Travers 2002), RT (DiNardo and Travers 1997), PBN (Yamamoto et al. 1994; Tokita et al. 2012) and GC (King 2018). All these studies examined one or a few brain areas associated with gustatory function, and therefore a complete picture of the number and distribution of neurons activated by different tastes is not available. Recently, several studies have used a new whole brain mapping approach to reveal networks and/or modules in the brain that may possess similar functionality (Bijoch et al. 2023; Oyaga et al. 2023; Stefaniuk et al. 2023; Aboharb et al. 2025). However, no study has attempted to document the number and location of neurons activated by IO infusions in all brain areas associated with gustatory function in the same subjects. Therefore, the current study was designed to produce a more extensive map of taste-activated neurons by examining multiple areas in the same animals.

Fos immunohistochemistry has been used successfully to label neurons activated by IO infusion of taste solutions in rats and mice (Yamamoto et al. 1994; Harrer and Travers 1996; DiNardo and Travers 1997; King et al. 1999, 2003, 2014; Riley and King 2013; King 2018; Boughter et al. 2019). Fos-immunoreactive (Fos-IR) neurons can be counted as a measure of the number of activated neurons and can be located to specific nuclei as well as their subareas. Therefore, the goal of our study was to map the neurons activated by IO infusion of a sweet (sucrose) and bitter (quinine hydrochloride) taste, as well as filtered water as a control, throughout the brains of C57Bl/6 mice. IO stimulation was used to try and provide a similar level (i.e., volume) of orosensory stimulation among tastes. Fos-IR neurons elicited by each treatment were counted in 29 taste-related nuclei and subareas. This approach allowed us to directly compare our results to previous studies using Fos immunohistochemistry to label active neurons in gustatory nuclei as well as to obtain a more whole-brain map of neurons that respond to sweet and bitter taste.

## Results

**Fig. 1:**
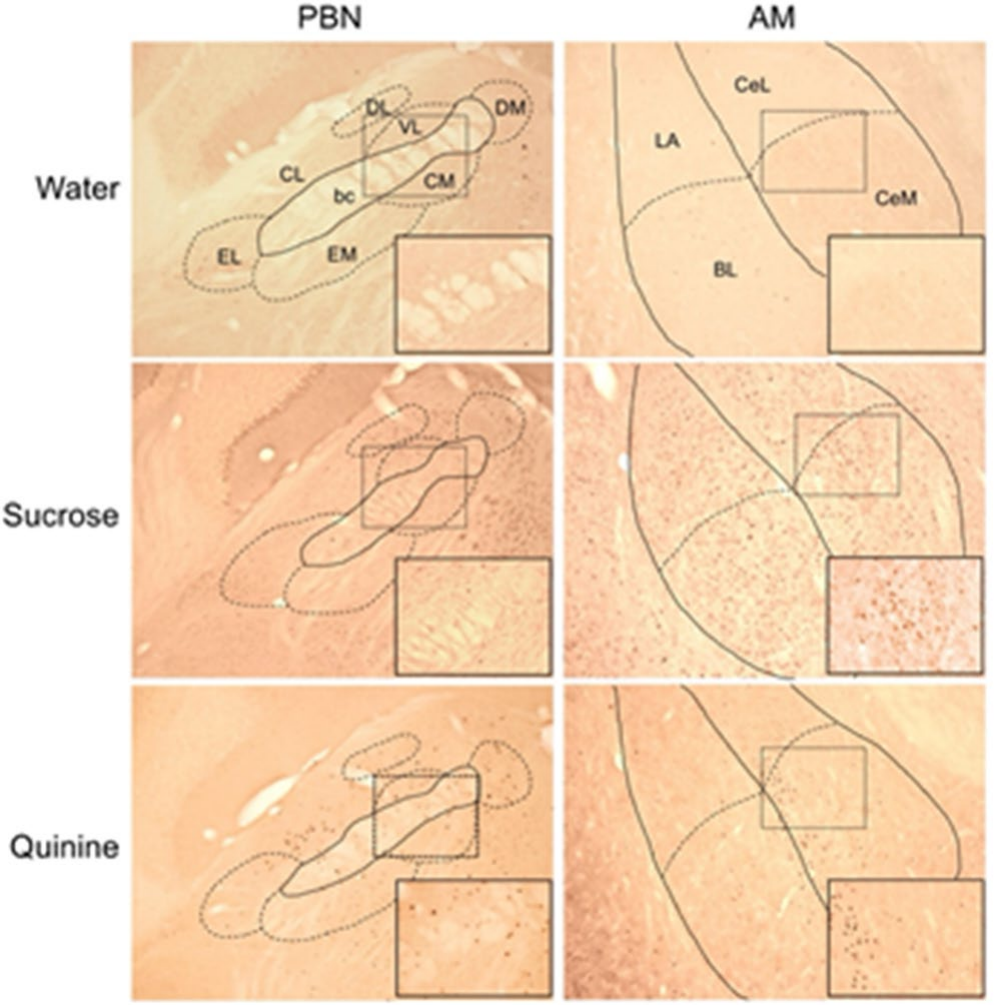
Images of the parabrachial nucleus (PBN; left) and amygdala (AM, right) showing Fos-IR neurons elicited by IO infusion of filtered water (top), 1.0 M sucrose (middle) or 3.0 mM quinine hydrochloride (bottom). Subnuclei are indicated using the abbreviations in Table 1 and bc is brachium conjunctivum. The scale bar at the lower left measures 0.5 mm for the main image and 0.125 mm for the inset. The area included in the inset is outlined with a dashed line in the main image

**Fig. 2:**
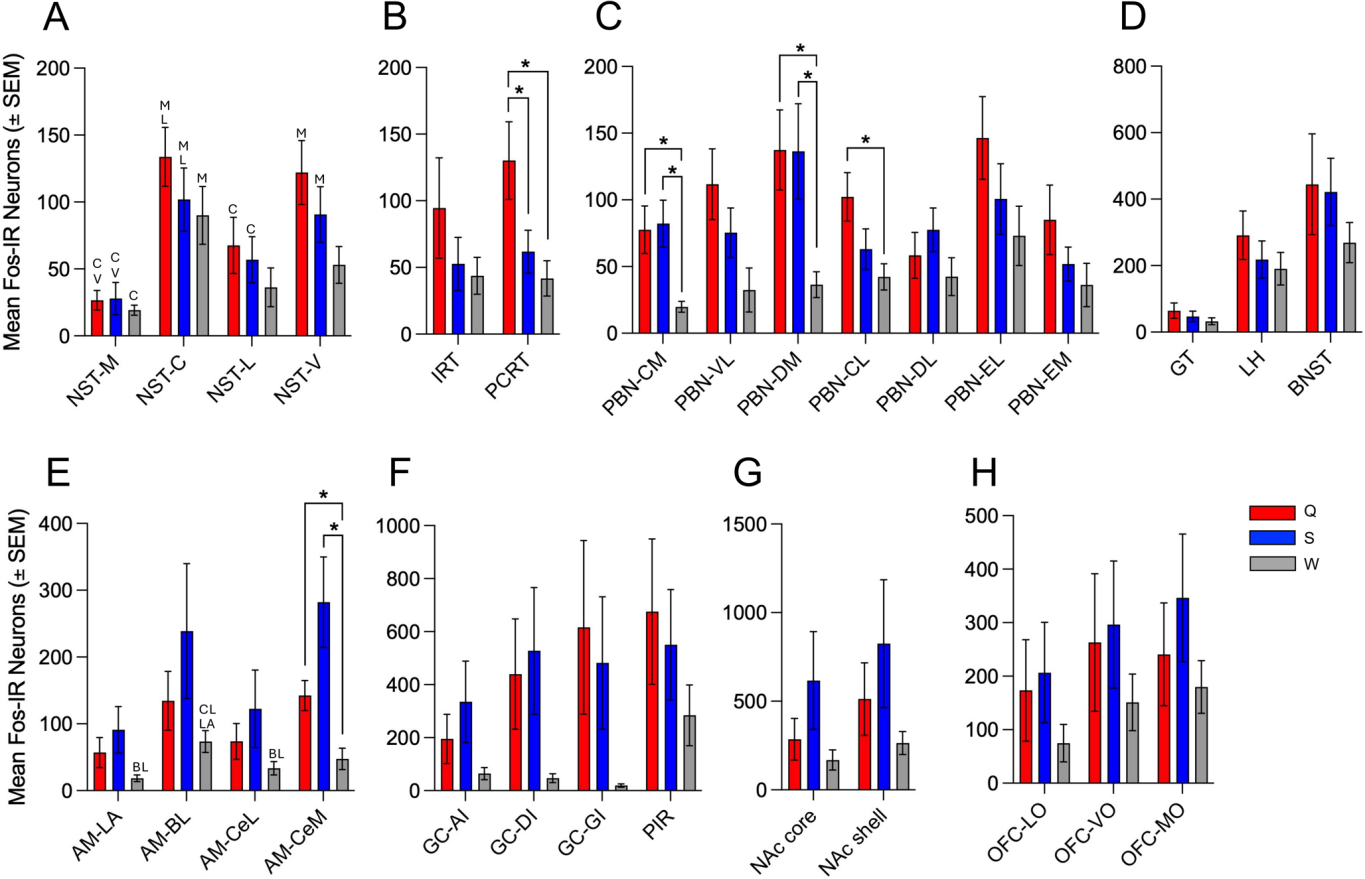
Figure 2. Graphs of the mean (± SEM) number of Fos-IR neurons in the NST (A), RT (B), PBN (C), GT, LH and BNST (D), AM (E), GC and PIR (F), NAc (G) and OFC (H) comparing the effects of IO infusion of 3.0 mM quinine hydrochloride (red bars), 1.0 M sucrose (blue bars) and filtered water (gray bars). *Indicates that there was a significant difference between the bars connected by brackets and letters indicate a significant difference between subareas of a nucleus that begin with those letters. Subnuclei abbreviations are as indicated in Table 1.

Fos-IR neurons were located in each nucleus and subarea examined following IO infusion of W, S or Q (Figs. 1 and 2). When counts of Fos-IR neurons were totaled across all brain regions in each mouse, IO delivery of Q (5904.2 ± 2010.9; mean ± SEM) or S (6586.8 ± 2386.6) tended to elicit more Fos-IR neurons than W (2484.8 ± 668.64). However, these overall differences were not significant (F [2,15] = 1.26; *p* = 0.32). Additionally, no significant differences between stimuli were found in either control area (Fs [2,15] ≤ 0.96; ps ≥ 0.41). In the brainstem, significant differences in the number of Fos-IR neurons among solutions were found in the PCRT and in the CM, DM and CL subareas of the PBN. In the PCRT, there were more Fos-IR neurons after IO infusion of Q compared to S and W (ps < 0.05; Fig. 2B). In CM and DM, both Q and S elicited more Fos-IR neurons than W, while in CL only the effect of Q was greater than W (ps < 0.05; Fig. 2C). The CM and VL, together with a small group of neurons that traverse the brachium (i.e., the superior cerebellar peduncle), comprise the primary gustatory area of the PBN. While the main effect of stimulus was not quite significant for the VL (*p* = 0.06), when counts from the CM, VL and brachium were combined there was a significant effect of stimulus (F [2,25] = 4.18; *p* = 0.04), with more Fos-IR neurons elicited by Q as compared to W (*p* = 0.01). Among forebrain areas, the only significant differences between stimuli were found in the CeM of the amygdala (AM). Here, there were more Fos-IR neurons following IO infusion of S or Q than W (*p* < 0.05, Fig. 2E).

We also compared the number of Fos-IR neurons in the subareas of a nucleus within treatment and found significant differences in the NST and AM. Within the NST, there were fewer labeled neurons in the anatomically smaller M than C subarea for all solutions (ps < 0.05; Fig. 2A). Also, in the NST, there were fewer Fos-IR neurons in M than V and more in C than L following IO infusion of Q and S but not W (*ps* < 0.05, Fig. 2A). In the AM, the only differences among subareas were following IO infusion of W where there were more labeled neurons in BL than LA and CeL (ps < 0.05; Fig. 2B). When comparing the location of Fos-IR neurons along the anterior-posterior extent of a nucleus, the only differences were in the NST and PBN with the caudal-most NST section having more labeled neurons elicited by IO infusion of Q than all other sections and the first or second caudal PBN sections having fewer labeled neurons following IO infusion of Q and S (ps < 0.05). There were no differences in the location of Fos-IR in any other subarea or section examined within treatment, including in the non-taste areas examined as controls (the IC and PV, data not shown).

**Fig. 3:**
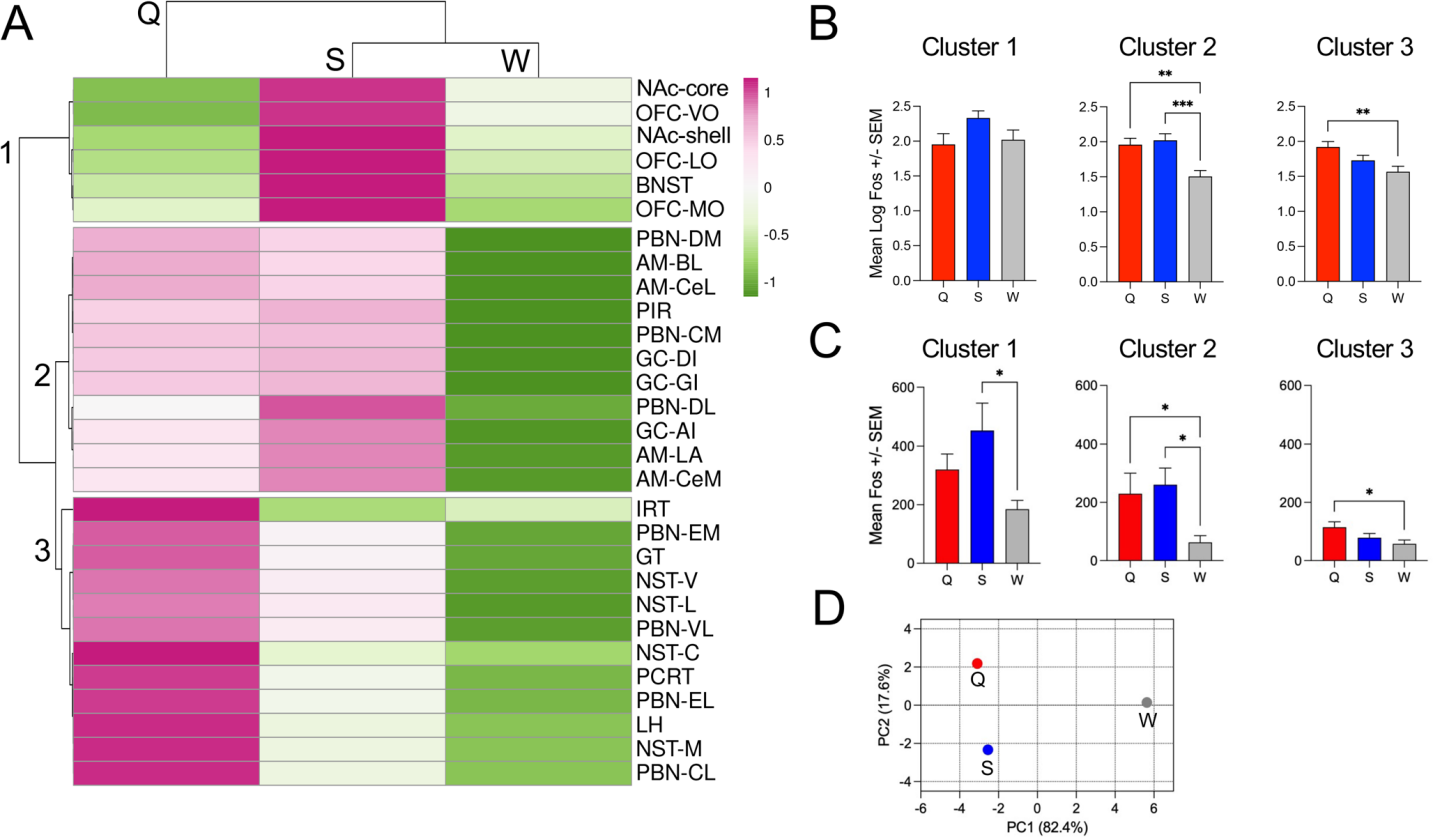
Hierarchical clustering and principal components analysis (PCA) of the mean number of Fos-IR neurons in brain areas according to stimulus. (A) Heatmap showing relative Fos expression values derived from log-transformed data, which were then converted to z-scores (calculated for each row/subarea). Scale: Pink indicates relatively higher expression and green relatively lower expression. Both rows and columns are clustered using correlation distance and average linkage. Three primary clusters are identified on the Y-axis. (B) Mean Fos-IR neuron counts, calculated from log-transformed data and combined according to cluster. (C) Mean Fos-IR neuron counts, calculated from log-transformed data and combined according to cluster. (D) Stimuli represented in 2-dimensional space according to PCA; Component 1 and 2 collectively account for > 99% of the total variance. *Indicates that there was a significant difference between the bars connected by brackets; *p < 0.05, **p < 0.01, ***p < 0.001

**Fig. 4:**
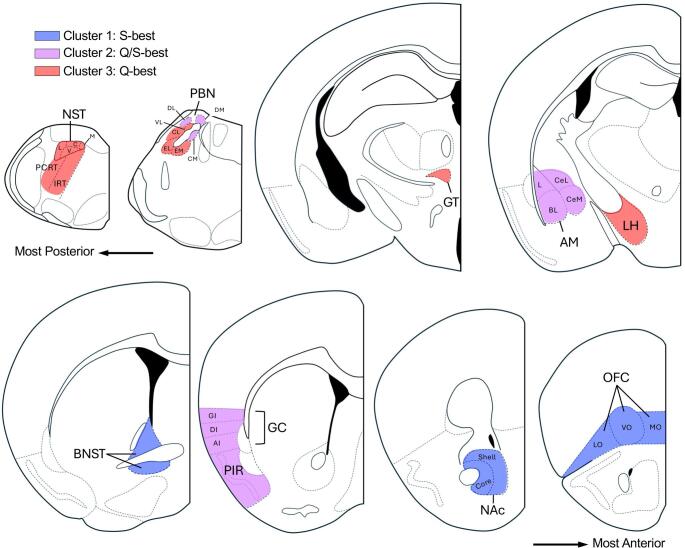
Results of the hierarchical cluster analysis depicted using representative sections of brain nuclei and their subareas. Coronal sections are arranged from most posterior (upper left) to most anterior (lower right). Areas responding relatively strongest to Q (red) were found more posteriorly, whereas those responding best to S (blue) were found anteriorly; areas responding to both quinine and sucrose (violet) better than W were distributed at more intermediate levels. Abbreviations are as in Table 1

To better determine potential commonalities in response to the 3 stimuli across all brain areas, we performed hierarchical cluster analysis (Fig. 3A). This analysis provided evidence that brain nuclei/subareas could be sorted into 3 principal clusters or modules based on correlation distance. This included a cluster of brain areas responding relatively best to S, a cluster responding well to either S or Q, but not W, and a third cluster responding best to Q. Although this analysis considers relative similarity rather than absolute differences in Fos-IR cell counts, when either log-transformed or raw mean Fos-IR counts for each area were combined into groups based on cluster assignment, comparison of taste stimuli still yielded corresponding significant differences (Fig. 3B-C). These distinct sensitivity groupings were further underlined by PCA, which suggested independence in the overall representation of the 3 stimuli across the brain areas surveyed (Fig. 3D). Consideration of the brain areas along the anterior-posterior axis by cluster (Fig. 4) revealed a pattern of sensitivity with Q sensitivity predominating in brainstem areas including NST, RF, and part of PBN. On the other hand, areas equally sensitive to Q and S included the AM, GC, PIR, as well as a few PBN subareas, while areas most sensitive to S were all anterior forebrain areas like the BNST, NAc and OFC.

## Discussion

In this study, we mapped the location of neurons throughout the B6 mouse brain that respond to IO infusion of Q, S or W using Fos immunohistochemistry. The goals were to describe the number of Fos-IR neurons elicited by each stimulus, and to determine if the effects of the three treatments were different. Previous studies using IO delivery of tastants have typically focused on one or just a few brain areas, especially the NST or PBN, and most used rats as subjects (Yamamoto et al. 1994; Harrer and Travers 1996; DiNardo and Travers 1997; King et al. 1999, 2003, 2014; Travers 2002; Riley and King 2013; Stratford and Thompson 2016; King 2018; Boughter et al. 2019; Yamamoto and Sawa 2000; Loney and Eckel 2021). Here, we examined a total of 29 different regions, including 12 brain nuclei and their subareas, reported to be involved in some aspect of taste processing in the same mice to get a more complete picture of the neuronal populations activated by IO infusions of a bitter and sweet tastant, as well as water. We found Fos-IR neurons in all nuclei and subareas examined following IO infusion of each stimulus. Significant differences in the number of Fos-IR neurons elicited by IO infusion of different solutions were found in the PCRT, several subareas of the PBN, and in one subarea of the amygdala. In addition, we identified clusters of nuclei that tended to respond best to Q and/or S than W.

Before discussing details of the data, it is important to consider the technical limitations of the current study and the reliability of the data. Although Fos immunoreactivity has been used to identify neurons activated by IO infusion of solutions in numerous studies (cited above), this technique may only label a subset of activated neurons (Dragunow and Faull 1989) and does not label inhibited neurons (Aparicio et al. 2022). Moreover, some brain regions show constitutive expression of Fos protein, making the discrimination of stimulus-evoked Fos-IR neurons difficult (Kovács 2008). Another issue is that the labeling for the Fos protein was variable among subjects in the same treatment group, and this variability limited the number of statistically significant differences. However, the amount of variability seen in the current study in particular brain regions is not inconsistent with other published studies of taste-evoked Fos-IR that used similar group sizes (*n* = 5–6; cf., DiNardo and Travers 1997; Chan et al. 2004; Chen et al. 2011; Lin et al. 2012; Tokita et al. 2014; King 2018). Interestingly, in our study within-group variation was higher in forebrain areas than in the brainstem, with the coefficient of variation (CV) for Fos counts being 0.81 in forebrain and 0.56 in brainstem (combined across sites and averaged across stimuli). This finding of greater variability in forebrain is consistent with other (non-taste) brain-wide Fos expression studies and is possibly due to the dependence on activity in these areas to level of stress, arousal or attention (e.g., Oyaga et al. 2023; Aboharb et al. 2025). Going along with this, taste-evoked Fos-IR neurons in some forebrain regions including GC and GT are reliably modulated by novelty, while such effects in NST or PBN are inconsistent (cf., Lin et al. 2012; Bamji-Stocke et al. 2018; Wiaderkiewicz and Reilly 2023).

Each nucleus and subarea examined in the current study contained Fos-IR neurons after each stimulus, including the IO infusion of W, suggesting that taste and orosensory input activates neurons in each of these areas. Differences between stimuli were found primarily in the brainstem, including in the PCRT where Q was a more effective stimulus than W, a finding which is consistent with previous studies. For example, DiNardo and Travers (1997) found greater numbers of Fos-IR neurons in rats activated by IO delivery of 3 mM Q in both the PCRT and IRT relative to 1.0 M S or W. They concluded that this greater activation in the reticular formation reflects the contribution of these areas to aversive oromotor responses that are elicited by Q, confirmed by a subsequent study that found that IXth nerve transections eliminated both oromotor responses and Fos-IR neurons in the RF in response to Q (King et al. 1999). In addition, Travers et al. (2007) noted (but did not quantify) robust Fos-IR in the reticular formation evoked by IO infusion of 3 mM Q in FVB/NJ or GAD-transgenic mice.

Several studies have examined Fos-IR neurons in the NST following intraoral delivery of tastes in the rat (e.g., Harrer and Travers 1996; King et al. 1999; 2014; Travers et al. 1999; Travers 2002; Chan et al. 2004). As a direct comparison to our study, Harrer and Travers (1996) found a tendency for 3 mM Q or 1.0 M S to elicit more Fos-IR neurons throughout the NST than water or an unstimulated control, but a significant difference was only found in the NST-C (RC in their paper) with more Fos-IR neurons elicited by either tastant compared to water or control. This subarea receives most of the input from peripheral taste nerves (Lundy and Norgren 2015). Although we did not find a significant effect of stimulus in the NST-C, or the other NST subareas, we found that infusion of Q or S but not W activated more neurons in that subarea than in the NST-L (which is primarily involved in orotactile processing; Travers and Norgren 1995). Consistent with the glossopharyngeal nerve being responsive to bitter and terminating primarily within the caudal part of the rostral NST (Frank 1991), we also found that the caudal-most NST section examined contained more Fos-IR neurons than the more rostral sections after infusion of Q (but not S or W).

In the PBN, we found significant effects of stimulus in several subareas, including the CM, DM and CL. These effects confirm results from our previous study with mice using the same stimuli (Boughter et al. 2019). Within the PBN, the waist area (composed primarily of the caudal CM and VL subareas; Fulwiler and Saper 1984) is the main site of termination of the ascending gustatory projection from the NST (Herbert et al. 1990) and contains many taste-responsive cells in rodents (Norgren and Pfaffmann 1975; Halsell and Travers 1997; Tokita and Boughter 2016). Therefore, this area should contain more neurons activated by IO infusion of tastants compared to water, which our results confirmed. The CL subarea of the PBN also contains taste-responsive neurons (Yamamoto et al. 1994) and the DM subarea is interconnected with the amygdala (Saper and Lowey 1980; Moga et al. 1990; Bernard et al. 1993; Tokita et al. 2010) that regulates PBN responses to tastes (Lundy and Norgren 2001, 2004; Li et al. 2002, 2005).

In the forebrain, we measured significantly more Fos-IR neurons evoked by Q or S as compared to water in the CeM supporting a role for this amygdala subnucleus in processing taste input. Also supporting such a role, the central amygdala (particularly CeM) receives input from *Satb2 +* taste neurons in the PBN (Jarvie et al. 2021) and sends projections back to the gustatory brainstem (van der Kooy et al. 1984; Tokita et al. 2009). In addition, projections from the central amygdala have been shown to alter taste-evoked neural responses in the brainstem (e.g., Li et al. 2002; 2005; Lundy and Norgren 2004; Tokita et al. 2004; Kang and Lundy 2010) as well as oromotor behavioral responses to IO infusion of tastants (Riley and King 2013).

Other areas in the forebrain, including the subareas of GC and OFC, had a tendency for greater levels of Fos-IR neurons evoked by Q or S relative to water, although differences in counts did not reach statistical significance. In a previous study with rats using IO delivery of tastes, King (2018) found a significant difference between Fos-IR neurons evoked by either 3 mM Q or 30 mM HCl versus water across the entirety of GC. However, no difference was found between water (or an unstimulated control) and 5 other stimuli, including either 0.1 or 1.0 M sucrose. Moreover, these limited effects of taste stimuli on Fos expression also were found when the analysis was restricted to the DI subarea, which is the (primary) site of taste input from the GT, and principal location of taste-responsive neurons (Lundy and Norgren 2015). A recent brain-wide Fos-mapping study comparing sucrose and cocaine as stimuli in mice found an elevation of Fos-IR neurons following a single sucrose drinking session in some regions/cortical layers of GC, but not in DI (Bijoch et al. 2023). Interestingly, the same study showed no sucrose-evoked elevation in Fos in the GT, as in our study. It is important to point out that water itself evokes significant activity in taste-responsive neurons of GC, and diverse taste stimuli (including water) are equally effective in evoking neuronal responses in this area in behaving mice (Chen et al. 2021; Staszko et al. 2002).

Across the brain areas assessed, especially those in the forebrain, a straightforward comparison of stimuli did not reveal many stimulus-specific differences, a finding consistent with the literature. Therefore, we used multivariate analysis to try to detect stimulus-based similarities and differences among brain regions, similar to those performed in whole-brain Fos mapping studies (e.g., Bijoch et al. 2023; Zimmerman et al. 2025). Hierarchical analysis identified 3 main clusters characterized by greater relative responsiveness to either Q, S, or both tastes compared to water. Elevated sensitivity to Q predominated in the brainstem, defining a group of structures including the NST, reticular formation (PCRT and IRT) and several regions of the PBN. This organization makes sense for the RT, as its subregions comprise part of the neuronal circuitry involved in motor taste reactivity, for which Q is a particularly potent stimulus (Travers et al. 1997; Travers 2002). In the NST, however, in vivo physiological recordings in rats or mice inevitably find that Q-best neurons are the least numerous type, with many more cells responding best to either NaCl or sucrose, even though bitter stimuli can still be potent neurophysiological activators of other taste cell types in this nucleus (e.g., Nakamura and Norgren 1991; Lemon and Smith 2005; McCaughey 2007). The inclusion of the PBN-EL with the Q group makes sense due to the concentration of CGRP-expressing neurons in this subarea that are responsive to diverse aversive or threatening stimuli, including bitter taste (Campos et al. 2018); these neurons are also found in other parts of the PBN. Less intuitive is the inclusion of the GT and LH in this group, although robust Q-evoked responses were found in both regions in electrophysiological studies (e.g., Li et al. 2013; Samuelsen et al. 2013). Moreover, there is recent evidence that certain subpopulations of neurons in the GT respond broadly to aversive stimuli, including Q (Cao et al. 2025).

Mismatches between c-Fos expression and physiological profiles in a given area among tastes are not easily explained (Travers 2002), and may be due to methodological differences, or to some interaction of cellular properties and methods. For example, IO Q may elicit c-Fos expression in cells that also respond to other tastes, or in cells that are unresponsive or difficult to record from using electrophysiological methods due to size, neuronal density, or other factors. A possible methodological factor may be the type of taste stimulation; in many neurophysiological studies with anesthetized animals, stimulation is mainly restricted to the anterior tongue and palate, whereas IO infusion may more effectively target the entire oral cavity (Grill and Norgren 1978). According to Geran and Travers (2009), the majority of bitter-best cells in either the NST or PBN have receptive fields in the posterior oral cavity, which may explain their underrepresentation in some in vivo studies, as taste buds in posterior regions are generally more difficult to stimulate.

A second cluster of brain areas was identified as similarly best-responsive to either Q or S, including GC, PIR, several subareas of the PBN and the 4 subareas of the amygdala, so taste-related processing in these areas may be linked to, or dependent upon, the presence of a stimulus, without the need to distinguish among them. On the other hand, there is much evidence that GC-AM neuronal circuitry is involved in encoding valence or hedonic character (e.g., Fontanini et al. 2009; Schiff et al. 2018; Wang et al. 2018), and stimuli representing hedonic extremes (S and Q) might both elicit robust activity in either of these areas, but in separate populations of cells. PIR is the primary olfactory cortex, yet there is ample evidence of taste-evoked activity there in animal and human studies (e.g., Small et al. 2004; Stapleton et al. 2006), especially in the context of taste-odor pairings. Bamji-Stocke et al. (2018) found Fos expression in both GC and PIR in rats evoked by either a taste (0.1 M sucrose), odor, or taste-odor mixture; although not compared with water, the expression of Fos-IR neurons in either area tended to be higher when the stimulus was novel vs. familiar.

The third cluster of brain regions was classified based on their relatively stronger response to S. These areas were found more anteriorly and included the BNST, NAc, and OFC. The inclusion of the latter two areas in this cluster makes senses as there is very strong evidence they are involved in reward processing, including reward evoked by or linked to sweet tastes. In the Bijoch et al. (2023) study, either novel or familiar sucrose increased Fos expression in NAc and oral delivery of sucrose in rats leads to dopamine release in NAc, even in the absence of post-ingestive signaling (Hajnal et al. 2004). OFC plays an important role in reward processing (reviewed in Wallis 2007; Noonan et al. 2012) and neurons in this cortical area that respond to sucrose (and other tastes) include those that encode sweet intensity (e.g., Furuyashiki et al. 2008; Fonseca et al. 2018). Stimulation of the BNST has been shown to modulate taste responses in the brainstem (Smith et al. 2005; Li and Cho 2006). Furthermore, inhibition of the BNST suppresses intake of sweet stimuli (but not other tastes; Kikuchi et al. 2023) and a recent study provides evidence both that neurons in the BNST respond to sweet tastes and that the BNST plays an integral role in a larger network controlling sweet-related consumption (Canovas et al. 2025). In fact, it is certainly possible that c-Fos expression in this study in particular regions may represent post-ingestive consequences of these stimuli, especially for sucrose (e.g., Yamamoto and Sawa 2000).

In conclusion, although the IO infusion of Q, S, or W elicited a similar number of Fos-IR neurons in specific nuclei and their subareas, different populations of neurons across all taste-responsive areas examined throughout the B6 mouse brain tended to respond best to Q or S, or both tastants, than to water. The cluster of brain areas relatively responsive to Q tended to be in the brainstem (NST, RT, and part of PBN) while the areas included in the S-best cluster were all in the forebrain (BNST, NAc, and OFC) and the areas that responded better to Q and S than W included some subareas of the PBN in the brainstem as well as the AM, GC, and PIR in the forebrain. Therefore, these data suggest a loosely regional distinction among areas responding best to Q and S and are consistent with the idea that multiple brain areas work together to identify if a taste or just water is being ingested, to determine if that taste is bitter or sweet and to participate in the appropriate aversive or ingestive responses.

## Methods

### Animals

16 adult C57BL/6J mice (7 males, 9 females) were tested in this experiment; ages ranged from 92 to 128 days. All animals were housed on a 12 h light/12 h dark cycle and group housed in standard plastic shoebox cages prior to being assigned to either experimental group. After assignment, mice were singly housed and chow (Teklad LM-485) and water were available *ad libitum* except on the testing day and part of the previous day (see below). All procedures were approved by the University of Tennessee Health Science Center Institutional Care and Use Committee.

### Surgery

All mice underwent a brief surgical procedure for the implantation of a unilateral IO cannula (Tokita et al. 2014; Boughter et al. 2019). Mice were anesthetized with i.p. injection of ketamine/xylazine (100/10 mg/kg) and positioned in a non-traumatic head holder. Body temperature was maintained at 35° C with a heating pad. The scalp was opened with a midline incision. Polyethylene tubing (PE 50) was inserted (with the aid of a 25-g needle) through the right buccal mucosa and led along the lateral surface of the skull; the oral end of the cannula was flared to prevent its recession and placed between the cheek and gum next to the first maxillary molar. The needle was removed, and the resulting cannula was secured to the exposed skull surface with dental cement and a small surgical screw. Training began the following day.

### Stimulation

Mice underwent a training/habituation procedure for 3 days prior to testing. During this procedure, they received a constant influx of filtered water through the IO cannula (0.1 ml/min for 15 min) using a precision syringe pump (model 341 A; Sage Instruments, Cambridge, MA, USA) in a round Plexiglas test chamber (11.5 cm diameter, 14.5 cm height). The following day, they were stimulated with a freshly prepared solution of 1.0 M sucrose (*n* = 6), 3.0 mM quinine hydrochloride (*n* = 5), or filtered water (*n* = 5) using the same parameters as the habituation. Stimuli and concentrations were selected based on previous studies in mice and rats (Dinardo and Travers 1997; King 2018; Boughter et al. 2019); stimulation parameters were the same as used in our previous IO c-Fos study in mice (Boughter et al. 2019), which were in turn based on those used by Stratford and Thompson (2016). Mice were deprived of both food and water 20 h prior to the test session, to help ensure the stomach was empty. Following testing, mice were placed in their home cage without additional food or water and were anesthetized and perfused at a time point corresponding to 2 h following the onset of testing (e.g., Tokita et al. 2014).

### Fos immunohistochemistry

Mice were perfused transcardially with 0.02 M phosphate-buffered saline (PBS) followed by ice cold 4% paraformaldehyde in 0.1 M phosphate buffer. Brains were extracted and postfixed for 24 h in the same medium, followed by cryoprotection in a 20/10% sucrose/glycerol solution in 0.1 M PB for at least 1 week. Brains were then frozen with dry ice, and serial coronal Sect.  (40 µm) cut on a freezing microtome. One series was Nissl-stained with cresyl violet (CV) to reveal cytoarchitecture, and the adjacent series was used for Fos immunoreactivity using the DAB (3, 3’-diaminobenzidine) method. Sections were rinsed with PBS 3 times, quenched with 3% hydrogen peroxide for 30 min, and washed 3 times in PBS again. Sections were incubated overnight with a rabbit anti-c-Fos primary antibody (1:5000, Santa Cruz Biotechnology, CA, USA) in PBS containing 3% normal donkey serum and 0.5% triton X-100. Following primary antibody incubation, sections were rinsed 3 times in PBS and incubated for 1 h with a biotinylated donkey anti-rabbit IgG (1:1000, Jackson ImmunoResearch, West Grove, PA, USA). Sections were rinsed 3 times in PBS and reacted in a DAB solution for 10 m. The reaction continued for another 10 m after H_2_O_2_ was added (14 µL to 5 mL DAB solution; ~0.003% H_2_O_2_), followed by a final rinsing 3 times in PBS. Sections were mounted in rostral-caudal order on silane-coated slides (P4981-001; Thermo Scientific, Waltham, MA, USA). Slides were dried for at least 24 h before CV staining or coverslipping. A few sections (taken from the series set aside for CV) of some of the mice were processed for immunohistochemistry as described above, except with the primary antibody omitted; these sections showed no discernable staining for Fos, as compared to antibody-incubated sections from the same level.

### Data collection and analysis

The total number of Fos-IR neurons were counted manually on the left side of 13 nuclei and their subareas (31 total regions) while looking through the oculars of a Zeiss Axioscope microscope at 200x magnification (counts were made by one of the investigators who was blind to the identity of the animal). Fos-IR neurons were identified as having brown- or black-immunostained nuclei that were distinct from background labeling. The focal plane was adjusted during counting to ensure that all labeled neurons were noticed, and each subarea was examined more than once to ensure that labeled neurons were not missed nor double counted. Before counting, images of sections to be examined were captured with an AxioCam MRc camera and ZEN Blue software (Carl Zeiss Inc) and shown on a computer screen adjacent to the microscope. The nuclei and subareas were drawn on these images with the aid of the mouse brain atlas of Paxinos and Franklin (2001) and referred to while counting Fos-IR neurons. Each of the 13 nuclei were examined in a different number of sections depending upon its rostral-caudal extent (Table 1). For the PBN, the CL, DL, EL, and EM subareas only appeared in the rostral 4 sections examined. As a control for potential variability in the immunohistochemical procedure, Fos-IR neurons were counted in the inferior colliculus (IC) and the paraventricular thalamic nucleus (PV).

To thoroughly describe the location of Fos-IR neurons following each treatment and to determine if the number and location of labeled neurons were different among treatment groups, the data were examined two ways. First, the raw counts of the number of Fos-IR neurons in each nucleus and subarea were used to describe the distribution of labeled cells following IO infusion of Q, S or W, as well as to determine if there were differences among these treatments. Second, the possible interrelationship of different brain nuclei/subareas to one another according to stimulus activation was examined using hierarchical cluster analysis and principal components analysis (PCA).

Raw count data for total brain counts and all subareas were examined for normality using the Shapiro-Wilk test. For inter-stimulus or within-area comparisons, homogeneity of variance was tested using the Brown-Forsyth test. 90% of raw data cells passed the normality test, and 80% of comparisons passed the test for homogeneity of variance. Based on these results, we proceeded cautiously with parametric testing, with the following caveat: all comparisons with unequal variance were re-tested following application of a log transform to the data, after which these passed the Brown-Forsyth test. For raw (or log-transformed) cell counts, each brain area or subarea was examined independently between or within stimulus using single-factor ANOVA followed by post-hoc Fisher’s Least Significance Difference (LSD) tests (in each case where the transformed data were used, statistical results did not differ from those obtained with the raw data).

Hierarchical cluster and principal components analysis (PCA) were performed using the online analysis toolkit at ClustViz (https://biit.cs.ut.ee/clustvis/; Metsalu and Vilo 2015). Data were log-transformed prior to analysis. PCA was carried out on log-transformed data using singular value decomposition (SVD) with unit variance scaling. For hierarchical clustering, pairwise distances were based on correlation, and average linkage was used.

## Data Availability

All data are available upon reasonable request.
